# Supplementary material for: Comparative genomics of bacteria from amphibian skin associated with inhibition of an amphibian fungal pathogen, Batrachochytrium dendrobatidis
Source: PeerJ. 2023 Aug 22;11:e15714. doi: 10.7717/peerj.15714 (PMC10452622; doi:10.7717/peerj.15714)
Supplement: Table S1 — The first two columns of the table contain the sequencing ID of each isolate, the number of base pairs and number of clusters that passed Illumina’s internal quality filtering procedure PF yield and number of PF clusters respectively. The other two columns contain the percentage of reads that had a Phred quality score above 30 (Q30%) and the average quality score of the raw reads. [file peerj-11-15714-s001.pdf]

Supplemental Table 1: Information regarding raw Illumina reads. The first two columns of the table contain the sequencing ID of each isolate, the number of base pairs and number of clusters that passed Illumina's internal quality filtering procedure PF yield and number of PF clusters respectively. The other two columns contain the percentage of reads that had a Phred quality score above 30 (Q30%) and the average quality score of the raw reads.

**Sequencing ID**	**PF* Yield (bp)**	**Number of PF* Clusters****	**Q30%**	**Average Quality Score**
LB-S02	1,952,360,238	6,464,769	91.33	38.14
LB-S04	1,954,006,440	6,470,220	91.3	38.13
LB-S05	1,709,751,558	5,661,429	91.7	38.23
LB-S06	1,782,628,386	5,902,743	91.68	38.23
LB-S07	1,715,350,940	5,679,970	91.46	38.17
LB-S08	1,681,662,538	5,568,419	91.77	38.25
LB-S09	2,140,794,346	7,088,723	90.84	38
LB-S10	2,250,450,848	7,451,824	90.81	37.99
LB-S11	2,031,885,294	6,728,097	90.86	38.01
LB-S12	1,982,936,530	6,566,015	91.09	38.07
LB-S13	2,086,145,634	6,907,767	91.21	38.11
LB-S14	2,012,697,120	6,664,560	91.17	38.1
LB-S15	2,096,819,522	6,943,111	91.14	38.08
LB-S16	2,047,650,600	6,780,300	91.38	38.15
LB-S20	2,138,597,598	7,081,449	90.07	37.81
LB-S21	2,314,181,606	7,662,853	90.25	37.85
LB-S22	2,313,662,166	7,661,133	90.55	37.94
LB-S23	2,261,351,538	7,487,919	90.34	37.88
LB-S27	1,991,684,564	6,594,982	88.79	37.45
LB-S28	2,049,137,044	6,785,222	89.03	37.51
LB-S29	2,040,933,818	6,758,059	88.94	37.49
LB-S30	2,156,068,298	7,139,299	87.97	37.21

LB-S31	2,112,098,910	6,993,705	87.49	37.08
LB-S32	2,123,267,776	7,030,688	88.35	37.32
LB-S33	2,618,246,078	8,669,689	89.02	37.51
LB-S34	2,191,960,696	7,258,148	90.48	37.9
LB-S35	2,112,356,214	6,994,557	90.63	37.94
LB-S36	2,100,396,108	6,954,954	91.19	38.09
LB-S37	2,206,094,900	7,304,950	91.06	38.06
LB-S38	1,961,188,302	6,494,001	91.03	38.05
LB-S39	2,297,436,008	7,607,404	90.1	37.81
LB-S41	2,250,771,572	7,452,886	90.49	37.91
LB-S42	2,603,546,832	8,621,016	89.11	37.53
LB-S43	2,408,450,906	7,975,003	89.6	37.67
LB-S44	2,488,300,008	8,239,404	89.26	37.58
LB-S45	2,666,098,280	8,828,140	89.35	37.6
LB-S46	2,696,225,800	8,927,900	89.32	37.59
LB-S47	2,618,102,628	8,669,214	89.71	37.7
LB-S48	2,451,845,588	8,118,694	89.4	37.62
LB-S50	2,230,779,776	7,386,688	88.58	37.4
