# Supplementary material for: Comparative genomics of bacteria from amphibian skin associated with inhibition of an amphibian fungal pathogen, Batrachochytrium dendrobatidis
Source: PeerJ. 2023 Aug 22;11:e15714. doi: 10.7717/peerj.15714 (PMC10452622; doi:10.7717/peerj.15714)
Supplement: Table S2 — The first four columns contain the bacterial genus/sequencing ID, how many contigs make up each genome (# contigs), the length in base pairs of the longest contig (Largest contig), and how many base pairs each genome contains (total length). The next five columns contain the percentage of each genome made up of G/C nucleotides (GC%), the length of the shortest contig that contains at least 50 or 75% of the genome (N50/N75) and the number of contigs that are needed to cover at least 50 or 75% of the genome (L50/L75). The final column contains the number of uncalled bases per 100 kilobase pairs (# N’s per 100 kbp). [file peerj-11-15714-s002.pdf]

Supplemental Table 2: Assembly statistics of whole bacterial genomes. The first four columns contain the bacterial genus/sequencing ID, how many contigs make up each genome (# contigs), the length in base pairs of the longest contig (Largest contig), and how many base pairs each genome contains (total length). The next five columns contain the percentage of each genome made up of G/C nucleotides (GC%), the length of the shortest contig that contains at least 50 or 75% of the genome (N50/N75) and the number of contigs that are needed to cover at least 50 or 75% of the genome (L50/L75). The final column contains the number of uncalled bases per 100 kilobase pairs (# N's per 100 kbp).

**Bacterial genus (Sample ID/Sequencing ID)**	**# contigs**	**Largest contig**	**Total length**	**GC (%)**	**N50**	**N75**	**L50**	**L75**	**# N's per 100 kbp**
Agrobacterium (HP11E/LB-S02)	56	688558	4680142	56.67	349712	166753	5	10	0
Agrobacterium (HP5J1/LB-S04)	45	1074136	4676747	56.69	348513	309462	4	7	0
Bacillus (CC6U/LB-S05)	81	1258992	6117020	37.4	1104946	113546	3	8	0
Bacillus (HP5F1/LB-S06)	119	445015	5627244	39.92	174213	85551	11	24	0
Bacillus (HP5K2/LB-S07)	120	445015	5628740	39.92	153687	84098	11	25	0
Bacillus (PRE21G/LB-S08)	113	1201680	6065909	37.44	918518	157784	3	7	0
Flavobacterium (CC8A2/LB-S09)	54	563445	5325413	34.01	286538	141230	7	13	0
Flavobacterium (HP3BB/LB-S10)	30	509329	4813557	33.78	365929	171784	6	11	0
Flavobacterium (HP3M/LB-S11)	29	509329	4813597	33.78	365927	171784	6	11	0
Hafnia (BFE23D/LB-S12)	70	466616	4734883	48.12	168785	100233	9	18	0

Hafnia (BFE501/LB-S13)	112	586722	5025182	47.89	180877	92853	7	18	0
Hafnia (PRE11D1/LB-S14)	124	538032	5022383	47.98	220892	79099	7	18	0
Hafnia (PRE7B/LB-S15)	123	538032	5022734	47.98	220892	79527	7	18	0
Hafnia (PRE7G/LB-S16)	123	538032	5022717	47.98	220892	79527	7	18	0
Janthinobacterium (CC11P/LB-S20)	68	1028192	6299659	63.1	448274	281560	5	10	0
Janthinobacterium (CC130/LB-S21)	211	284967	7734570	65.34	121673	68881	22	43	0
Janthinobacterium (HP12P2/LB-S22)	82	822193	6045046	62.61	296923	136953	6	14	0
Janthinobacterium (CC14D/LB-S23)	45	1336112	6263012	63.09	587332	275417	4	7	0
Massilia (HP1M/LB-S27)	63	1061463	5742643	63.18	420963	226562	4	9	0
Massilia (HP4P/LB-S28)	40	794939	5160980	64.14	274418	157776	6	12	0
Massilia (HP6N/LB-S29)	44	726155	5491525	63.59	220857	179845	6	13	0
Microbacterium (CC8G/LB-S30)	11	903157	3750929	68.3	844452	601843	3	4	0
Microbacterium (CC3Q/LB-S31)	14	970861	3658083	69.98	543564	401990	3	5	0
Microbacterium (HP3T/LB-S32)	14	635353	2846619	69.4	418522	337690	3	5	0
Microbacterium (HP4V/LB-S33)	26	756650	3854063	68.94	435233	299231	4	6	0
Pedobacter (HP10H/LB-S34)	118	1207930	7729651	41.75	306829	195337	6	14	0

Pedobacter (HP3C/LB-S35)	139	284735	5974156	38.41	91377	45790	19	41	0
Pedobacter (HP3Q1/LB-S36)	96	438734	5124477	38.09	134171	70880	11	24	0
Pedobacter (HP3S/LB-S37)	132	633395	5175166	39.08	331795	161592	6	12	0
Pedobacter (HP6J/LB-S38)	80	636287	5365052	38.66	213539	150054	8	16	0
Pseudomonas (CC7Q/LB-S39)	27	1296146	5900904	62.26	796000	349372	3	6	0
Serratia (CC9C/LB-S41)	34	512688	4656537	54.02	305391	229367	6	10	0
Sphingomonas (HP4T/LB-S42)	25	714119	4064073	66.37	410677	169571	4	8	0
Sphingomonas (HP5L/LB-S43)	13	1159454	3755240	67.37	782816	339697	2	4	0
Sphingomonas (HP7F/LB-S44)	15	1531233	4237281	67.67	743928	209334	2	5	0
Sphingomonas (HP7Q/LB-S45)	27	754655	4255872	66.14	455590	160645	4	8	0
Sphingomonas (HP9M/LB-S46)	41	496844	4036398	66.43	206638	112508	7	14	0
Stenotrophomonas (CC4G1/LB-S47)	19	1581745	4211189	66.88	457696	391294	3	5	0
Streptomyces (CC3C/LB-S48)	88	1022433	8897001	71.47	284754	122446	10	21	0
Streptomyces (CC6O/LB-S50)	98	524522	8517904	72.11	185866	116188	14	29	0
