# Supplementary material for: Comparative genomics of bacteria from amphibian skin associated with inhibition of an amphibian fungal pathogen, Batrachochytrium dendrobatidis
Source: PeerJ. 2023 Aug 22;11:e15714. doi: 10.7717/peerj.15714 (PMC10452622; doi:10.7717/peerj.15714)
Supplement: Table S3 — Bd inhibition values range from 100 (total inhibition of Bd growth in in vitro assay), to 0 (no inhibition), with a few negative values indicative of potential Bd growth facilitation. Table contains the genus/sequence ID, Bd inhibition score as well as the amphibian host. The fourth column contains information about the presence of predicted terpene (T), siderophore (S) and type III polyketide synthase (P) biosynthetic gene clusters (BGCs). If the isolate did not contain a terpene, siderophore or type III polyketide synthase BGC the cell was given a value of NA. [file peerj-11-15714-s003.pdf]

Supplemental Table 3: Summary of 40 bacterial isolates from amphibian skin. Bd inhibition values range from 100 (total inhibition of Bd growth in *in vitro* assay), to 0 (no inhibition), with a few negative values indicative of potential Bd growth facilitation. Table contains the genus/sequence ID, Bd inhibition score as well as the amphibian host. The fourth column contains information about the presence of predicted terpene (T), siderophore (S) and type III polyketide synthase (P) biosynthetic gene clusters (BGCs). If the isolate did not contain a terpene, siderophore or type III polyketide synthase BGC the cell was given a value of NA.

**Bacterial genus (Sample ID/Sequencing ID)**	**Bd Inhibition**	**Amphibian host**	**BGCs**
Agrobacterium (HP11E/LB-S02)	91	Spring Peeper	T/S
Agrobacterium (HP5J1/LB-S04)	10	Spring Peeper	T/S
Bacillus (CC6U/LB-S05)	8	American Toad	T/S/P
Bacillus (HP5F1/LB-S06)	90	Spring Peeper	T/S/P
Bacillus (HP5K2/LB-S07)	15	Spring Peeper	T/S/P
Bacillus (PRE21G/LB-S08)	-8	Eastern Newt	T/S/P
Flavobacterium (CC8A2/LB-S09)	58	American Toad	T/P
Flavobacterium (HP3BB/LB-S10)	62	Spring Peeper	T
Flavobacterium (HP3M/LB-S11)	92	Spring Peeper	T
Hafnia (BFE23D/LB-S12)	85	Bullfrog	S
Hafnia (BFE501/LB-S13)	94	Bullfrog	S

Hafnia (PRE11D1/LB-S14)	82	Eastern Newt	S
Hafnia (PRE7B/LB-S15)	94	Eastern Newt	S
Hafnia (PRE7G/LB-S16)	99	Eastern Newt	S
Janthinobacterium (CC11P/LB-S20)	98	American Toad	T
Janthinobacterium (CC130/LB-S21)	57	American Toad	T/S
Janthinobacterium (HP12P2/LB-S22)	100	Spring Peeper	T
Janthinobacterium (CC14D/LB-S23)	36	American Toad	T
Massilia (HP1M/LB-S27)	100	Spring Peeper	T
Massilia (HP4P/LB-S28)	96	Spring Peeper	T
Massilia (HP6N/LB-S29)	83	Spring Peeper	T/P
Microbacterium (CC8G/LB-S30)	-21	American Toad	T/P
Microbacterium (CC3Q/LB-S31)	4	American Toad	T/P
Microbacterium (HP3T/LB-S32)	100	Spring Peeper	T/P
Microbacterium (HP4V/LB-S33)	97	Spring Peeper	T/P
Pedobacter (HP10H/LB-S34)	81	Spring Peeper	T/P
Pedobacter (HP3C/LB-S35)	-2	Spring Peeper	T/S/P

Pedobacter (HP3Q1/LB-S36)	6	Spring Peeper	T/P
Pedobacter (HP3S/LB-S37)	-18	Spring Peeper	T/S/P
Pedobacter (HP6J/LB-S38)	100	Spring Peeper	T/S/P
Pseudomonas (CC7Q/LB-S39)	51	American Toad	NA
Serratia (CC9C/LB-S41)	81	American Toad	T
Sphingomonas (HP4T/LB-S42)	-2	Spring Peeper	T/P
Sphingomonas (HP5L/LB-S43)	-9	Spring Peeper	T/P
Sphingomonas (HP7F/LB-S44)	93	Spring Peeper	T/P
Sphingomonas (HP7Q/LB-S45)	-30	Spring Peeper	T/P
Sphingomonas (HP9M/LB-S46)	-11	Spring Peeper	T/P
Stenotrophomonas (CC4G1/LB-S47)	73	American Toad	NA
Streptomyces (CC3C/LB-S48)	1	American Toad	T/S/P
Streptomyces (CC6O/LB-S50)	31	American Toad	T/S/P
