# Supplementary material for: Comparative genomics of bacteria from amphibian skin associated with inhibition of an amphibian fungal pathogen, Batrachochytrium dendrobatidis
Source: PeerJ. 2023 Aug 22;11:e15714. doi: 10.7717/peerj.15714 (PMC10452622; doi:10.7717/peerj.15714)
Supplement: Figure S3 — These Sphingomonas isolates were isolated from amphibian skin. Bd inhibition values were obtained from in vitro challenge assays (Walke et al., 2017). The inhibition values refer to the mean inhibition. 100 = complete Bd inhibition (blue), 0 = no inhibition (yellow). For the purposes of this figure, isolates with negative scores indicating potential facilitation of growth (N=4) were set at zero. Isolates are labeled with genus and sequencing ID number. (Table S3) [file peerj-11-15714-s006.pdf]

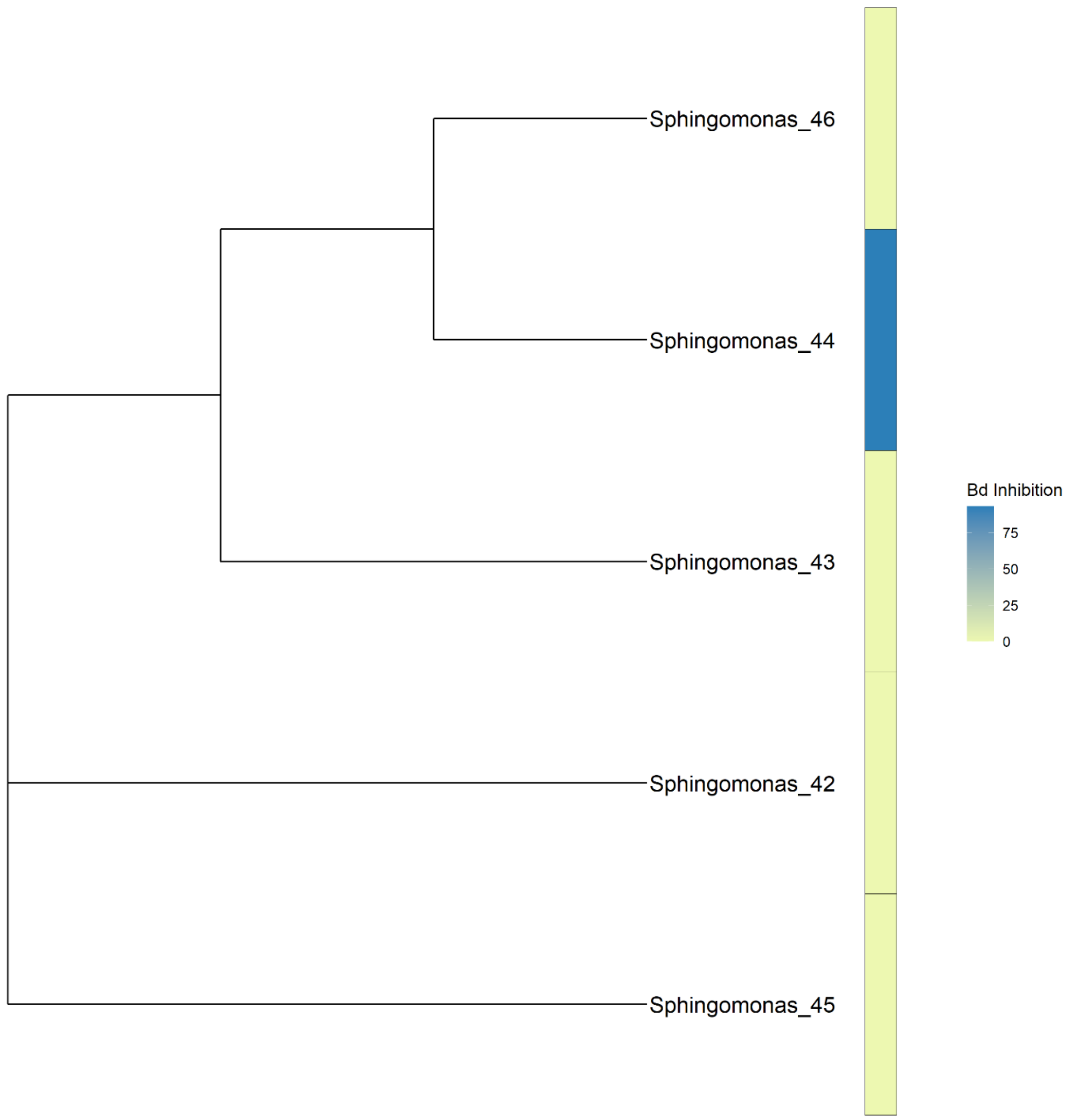

Fig. S3 Phylogenetic tree constructed from the eight genes that our five *Sphingomonas* isolates shared within their predicted Type III polyketide synthase cluster. These *Sphingomonas* isolates were isolated from amphibian skin. Bd inhibition values were obtained from in vitro challenge assays (Walke et al. 2017). The inhibition values refer to the mean inhibition. 100 = complete Bd inhibition (blue), 0 = no inhibition (yellow). For the purposes of this figure, isolates with negative scores indicating potential facilitation of growth (N=4) were set at zero. Isolates are labeled with genus and sequencing ID number. (Table S3)
